# Functional MRI for characterization of renal perfusion impairment and edema formation due to acute kidney injury in different mouse strains

**DOI:** 10.1371/journal.pone.0173248

**Published:** 2017-03-20

**Authors:** Susanne Tewes, Faikah Gueler, Rongjun Chen, Marcel Gutberlet, Mi-Sun Jang, Martin Meier, Michael Mengel, Dagmar Hartung, Frank Wacker, Song Rong, Katja Hueper

**Affiliations:** 1 Diagnostic and Interventional Radiology, Hannover Medical School, Hannover, Germany; 2 Nephrology, Hannover Medical School, Hannover, Germany; 3 Laboratory Animal Science, Hannover Medical School, Hannover, Germany; 4 Department of Laboratory Medicine & Pathology, University of Alberta, Edmonton, Canada; 5 The Transplantation Center of the affiliated hospital, Zunyi Medical College, Zunyi, China; UCL Institute of Child Health, UNITED KINGDOM

## Abstract

**Purpose:**

The purpose was to characterize acute kidney injury (AKI) in C57BL/6 (B6)- and 129/Sv (Sv)-mice by noninvasive measurement of renal perfusion and tissue edema using functional MRI.

**Methods:**

Different severities of AKI were induced in B6- and Sv-mice by renal ischemia reperfusion injury (IRI). Unilateral clamping of the renal pedicle for 35 min (moderate AKI) or 45 min (severe AKI) was done. MRI (7-Tesla) was performed 1, 7 and 28 days after surgery using a flow alternating inversion recovery (FAIR) arterial spin labeling (ASL) sequence. Maps of perfusion and T1-relaxation time were calculated. Relative MRI-parameters of the IRI kidney compared to the contralateral not-clipped kidney were compared between AKI severities and between mouse strains using unpaired t-tests. In addition, fibrosis was assessed by Masson Trichrome and collagen IV staining.

**Results:**

After moderate AKI relative perfusion impairment was significantly higher in B6- than in Sv-mice at d7 (55±7% vs. 82±8%, p<0.05) and d28 (76±7% vs. 102±3%, p<0.01). T1-values increased in the early phase after AKI in both mouse strains. T1-increase was more severe after prolonged ischemia times of 45 min compared to 35 min in both mouse strains, measured in the renal cortex and outer stripe of outer medulla. Kidney volume loss (compared to the contralateral kidney) occurred already after 7 days but proceeded markedly towards 4 weeks in severe AKI. Early renal perfusion impairment was predictive for later kidney volume loss. The progression to chronic kidney disease (CKD) in the severe AKI model was similar in both mouse strains as revealed by histology.

**Conclusion:**

Quantification of renal perfusion and tissue edema by functional MRI allows characterization of strain differences upon AKI. Renal perfusion impairment was stronger in B6- compared to Sv-animals following moderate AKI. Prolonged ischemia times were associated with more severe perfusion impairment and edema formation in the early phase and progression to CKD within 4 weeks of observation.

## Introduction

Inbred mouse strains are commonly used in experimental studies, as they are known to serve as robust models to mimic human disease. The development of transgenic and knock-out mice to investigate effects of single genes on disease progression has even led to increased use of mouse models. However, differences in genetic background might influence the result of an experimental study [1]. Different outcomes among mouse strains have been discovered in animal models of renal injury, hypertension, ischemia reperfusion injury (IRI) of the brain and the kidney and in many more disease models [1–7]. Thus, the conclusion of a trial may be influenced by the strain of the animal.

Renal IRI contributes to acute kidney injury (AKI) in many clinical settings [8,9]. It often occurs after major surgery (e.g. cardiothoracic or trauma) or in patients with hemodynamic instability [10,11]. Depending on the severity, AKI can lead to poor outcome and increased mortality and progression to chronic kidney disease (CKD) [12]. Decreased renal perfusion and inflammation with tissue edema after IRI leads to not only AKI but also to progressive renal fibrosis as a long-term consequence [13–15].

A recent study described differences in the susceptibility to unilateral IRI between B6- and Sv-mice when comparing inulin clearance to measure glomerular filtration rate, and immunohistochemistry [4]. However, these measurements were endpoint measurements and did not allow longitudinal follow-up. Functional MRI however, allows noninvasive detection and quantification of renal abnormalities associated with AKI in a longitudinal setting repetitively in the same individual mice [16–18]. Arterial spin labeling (ASL) has been shown to be reliable for the quantitation of renal perfusion impairment [16,19] and T1-mapping to measure the tissue water content which enhances due to increased capillary leakage after IRI [17].

The purpose of this study was to characterize renal perfusion impairment and tissue edema formation after renal IRI in B6- and Sv-mice by noninvasive functional MRI.

## Material and methods

### Animals

All experiments were performed with male C57BL/6 J^Han-ztm^ (B6)- and male 129/Sv- S1 (Sv-)-mice (12–16 weeks, 20–30g) purchased from the institute of laboratory animal sciences (Hannover Medical School). Animals were cared for in accordance with our institutions and international guidelines for experimental animal welfare and all experiments had been approved by the local animal protection committee (Niedersächsisches Landesamt für Verbraucherschutz und Lebensmittelsicherheit (LAVES), approval number 11/0492). Mice were housed under conventional conditions with a 10/14 hour light/dark cycle, and had free access to food (Altromin 1324 standard mouse diet) and domestic quality drinking water. After surgery mice were monitored daily for physical condition and wellbeing. Termination criteria were decreased uptake of food and water, obvious inactivity or elevation of renal function parameters. However, since IRI was done unilateral no creatinine elevation was expected in this model.

### Renal ischemia reperfusion injury (IRI)

IRI was induced by transient unilateral clamping of one renal pedicle for either 35 min (moderate AKI) in B6- (n = 10) and Sv- (n = 9) mice or 45 min (severe AKI) in B6- (n = 7) and Sv-mice (n = 5), as described previously [4]. Duration of transient clamping of the renal pedicle has been defined according to previous studies: 35 min IRI resulted in almost complete tubular regeneration [17], restitution of tissue edema (measured by T2 mapping) [18] and returning of renal perfusion nearly to baseline [16] and was therefore defined as moderate AKI. 45 min IRI was associated with persistent tubular atrophy [17] and persistent impairment of renal perfusion [16] and was therefore defined as severe AKI. Briefly, mice were anesthetized with isoflurane (3% induction and 1,5–2% maintenance) and butorphanol was given as analgetic treatment. Median laparotomy was performed and a non-traumatic vascular clamp was applied unilateral to the renal pedicle for 35 or 45 min. After releasing the clamp, reperfusion was controlled by visual inspection. After the surgery mice were returned into their cages and monitored until they were fully awake. At the endpoint after 28 days of follow up mice were sacrificed by deep anesthesia and total body perfusion with ice cold saline solution causing circulatory arrest. Kidneys were retrieved for further work-up.

### Histology and immunohistochemistry

Kidneys were fixed in 4% paraformaldehyde and embedded in paraffin. Two μm sections were cut.

#### Fibrosis

Masson Trichrome was stained according to standard protocols in 5/9 Sv-mice and 9/10 B6-mice after moderate AKI and in 5/5 Sv-mice and 6/7 B6-mice after severe AKI. Semiquantitative scoring of interstitial fibrosis and tubular atrophy was done by an investigator blinded to the animal group assignment with more than 15 years experience: 0 = normal morphology i.e. no IFTA, 1 = 5–25% of the area affected i.e. mild IFTA, 2 = 25–50% i.e. moderate IFTA, 3 = 50–75% i.e. severe IFTA, 4>75% i.e. very severe IFTA. Collagen IV was stained by immunofluorescent labelling techniques on paraffin embedded tissue (Collagen IV primary antibody from Biozol # 1340-01 with 1:50 dilution; secondary antibody from Thermo Scientific Alexa-fluor 488 1:500 dilution) as described previously [20] in 6/9 Sv-mice and 7/10 B6-mice after moderate AKI and in 3/5 Sv-mice and 6/7 B6-mice after severe AKI. A scoring system was used for collagen IV deposition ranging from mild to severe (1–4).

#### Inflammation

Macrophages were visualized by F4/80 (Biolegend #122602, 1:200) and T-lymphocytes by CD3 (eBioscience #2C11, 1:200) staining in 7/9 Sv-mice and 6/10 B6-mice after moderate AKI and in 5/5 Sv-mice and 7/7 B6-mice after severe AKI. Interstitial infiltrates were scored semiquantitatively: 0: < 5 cells per view field, 1: mild infiltrates, 2: moderate infiltrates, 3: severe infiltration, 4: very severe infiltration.

### Functional MRI

In total, 31 animals underwent MRI on day 1, day 7 and day 28 after IRI.

The MRI examination was performed as described previously by Hueper et al. [16,17]. Briefly, a 7-Tesla animal scanner (Bruker, Pharmascan, Ettlingen, Germany) was used in combination with a one-channel circular polarized volume coil (Bruker, Ettlingen, Germany). Animals were anesthetized by isoflurane inhalation (5% induction, 2% maintenance). For the quantification of kidney volume, respiratory triggered, fat-saturated T2-weighted turbo spin echo (TSE) sequences were acquired in axial and coronal planes, covering both kidneys. The coronal plane was adjusted to the long axis of both kidneys. Scan parameters were: TR/TE = 1500/33 ms, averages = 2, matrix = 256x256, field of view = 35x35 mm^2^, slice thickness = 1 mm. For functional imaging (renal perfusion and T1-mapping), a fat-saturated flow-alternating inversion-recovery (FAIR) ASL-sequence with an echo-planar readout was performed in a central coronal plane by using the following parameters: TR/TE = 18000/16.4 ms, inversion times = 30, 100, 200, 300, 500, 700, 1000, 1200, 1500, 2000, 3000, 5000 and 8000 ms, matrix = 128x128, field of view = 35x35 mm^2^, section thickness = 2mm, number of sections = 1.

### Image analysis

To correct for respiratory motion the ASL-images were co-registered and parameter maps of T1-values and renal perfusion were calculated using the Matlab 2012b software (MathWorks, USA) as described earlier [16,17].

Mean T1-values and renal perfusion were determined separately for the ischemic and the non-ischemic kidney by one reader, who was blinded to the animal group identity. Regions of interest (ROI) were placed manually into the cortex, the outer stripe of the outer medulla (OSOM) and the inner stripe of the outer medulla (ISOM) of T1-maps and into the renal cortex of perfusion maps. Relative T1-values and relative renal perfusion were calculated as the percentage of the ischemic kidney compared to the contralateral, non-ischemic kidney at the same time point.

Total kidney volume was determined separately for each kidney by manual segmentation of the kidney on axial T2-weighted images. These analyses were performed with Osirix software (Pixmeo, Switzerland).

### Statistical analysis

For statistical analysis, SPSS 24 software (IBM Corporation, United States) was used. Data are presented as mean ± standard error of the mean (SEM). Comparison of the two mouse strains and the different AKI severities after 35 and 45 min IRI was carried out using the unpaired t-test. Changes of relative perfusion and relative T1-values over time were analyzed performing repeated measures ANOVA using adjustment for multiple comparisons with the Bonferroni method. Additionally, the correlation of relative T1-values and relative renal perfusion in the early period after AKI with relative kidney volume at day 28 and histology were evaluated with linear regression and the Pearson coefficient of correlation. Histological differences between groups were assessed by unpaired t-tests.

P-values less than 0.05 were considered to indicate a significant difference.

## Results

### Renal perfusion

**Fig 1:**
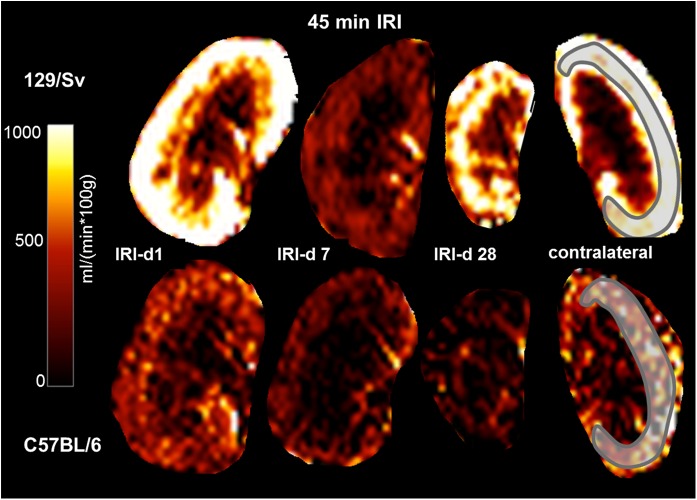
Perfusion-maps. Perfusion-maps after 45 min IRI for Sv- (upper row) and B6-mice (lower row) at d1, 7 and 28 are shown. The scheme depicts how the ROI was placed for quantification of cortical perfusion (in the contralateral, not clipped kidney). Renal perfusion impairment was most pronounced on day 7. Perfusion levels in the contralateral, not-clipped kidney were higher in Sv- than in B6-mice, suggesting a higher baseline perfusion.

For perfusion measurements the relative differences between the ischemic kidney and the contralateral non-ischemic kidney are given at the individual time points. An example of perfusion maps is shown in Fig 1. When comparing perfusion values of the contralateral, non-ischemic kidney (after both, moderate and severe AKI), perfusion levels of Sv-mice were higher at all time points (day 1: Sv 838±15 vs. B6 573±20 ml/min/100g, p<0.001; day 7: Sv 834±28 vs. B6 485±26 ml/min/100g, p<0.001; day 28: Sv 898±20 vs B6 505±27 ml/min/100g, p<0.001), suggesting higher levels of baseline perfusion in Sv-mice.

**Fig 2:**
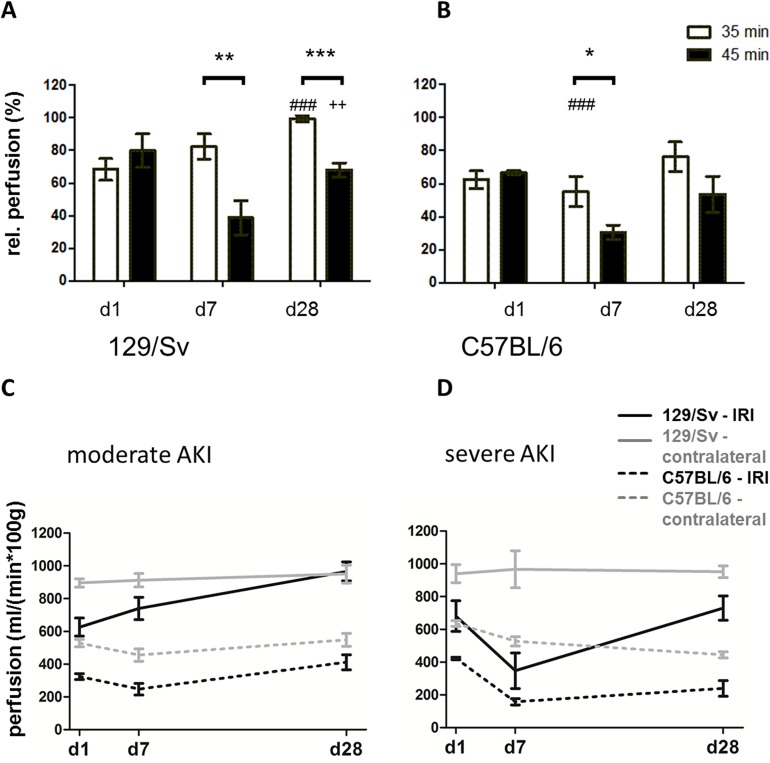
Comparison of perfusion measurements. Comparison of relative perfusion measured by ASL after moderate (white bars) vs. severe (black bars) AKI on day 1, 7 and 28 after IRI in Sv- (A) and B6- mice (B). Reduction of relative perfusion was most prominent in severe AKI (45min IRI, black bars). * = p<0.05, ** = p<0.01, *** = p<0.001. Differences over time, determined by ANOVA for repeated measurements, are presented as follows: ### = p<0.001 vs. day 1; ++ = p<0.01 vs. day 7. Absolute values of renal perfusion (ml/(min*100g)) are presented in Fig 2C and D. Values of the IRI-kidney are presented in black, values of the contralateral kidney are presented in grey. Differences between Sv-mice (continous lines) and B6 mice (dotted lines) were seen in moderate AKI (C) at day 7 and day 28 (D).

After moderate AKI, Sv-mice showed lowest relative perfusion on day 1, partial recovery on day 7 and normal values on day 28 with 102±3%. In contrast, after severe AKI, lowest values were observed on day 7 with permanent impairment at day 28 after AKI. In B6-mice, lowest perfusion values were observed on day 7 for both, moderate and severe AKI. Recovery of renal perfusion was seen on day 28 after moderate, but not after severe AKI (Fig 2).

When comparing renal perfusion in Sv-mice with different AKI severities we observed significantly lower renal perfusion values after severe compared to moderate AKI on day 7 and on day 28 after IRI (day 7: 82±8% (IRI 35min) vs. 33±8% (IRI 45min), p<0.01, day 28: 102±3% (IRI 35min) vs. 76±5% (IRI 45min), p<0.001). In B6-mice, however, renal perfusion impairment after severe AKI was aggravated compared to moderate AKI only at day 7 (day 7: 55±7% (IRI 35min) vs. 31±4% (IRI 45min), p<0.05) at day 28 no significance was reached (Fig 2A and 2B).

In the comparison between the two strains (Fig 2C and 2D), impairment of renal perfusion was more pronounced in B6-animals after moderate AKI: relative perfusion was significantly lower in B6- than in Sv-mice at d7 (55±7% (B6) vs. 82±8% (Sv; dotted line), p<0.05) and d28 (76±7% vs. 102±3%, p<0.01). No significant renal perfusion difference between the two mouse strains was observed after severe AKI (Fig 2D).

### Tissue edema

**Fig 3:**
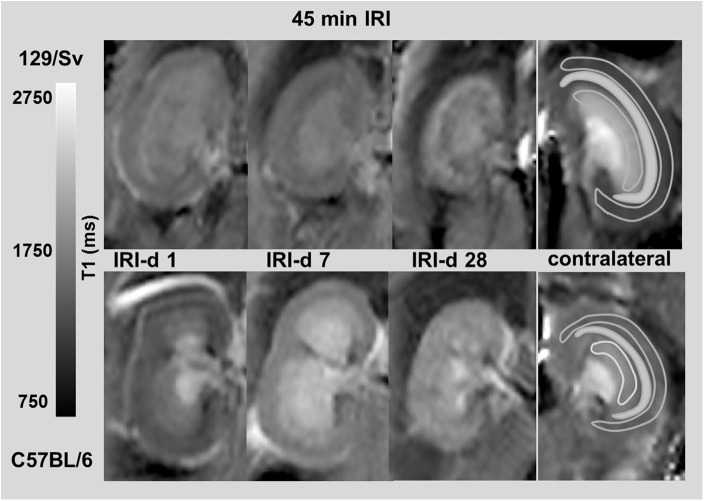
T1-maps. T1-maps after 45 min IRI for Sv- (upper row) and B6-mice (lower row) at d1, 7 and 28 are shown. In T1-maps spatial differences can be measured by placing ROIs in the cortex, OSOM and ISOM, which is illustrated in the contralateral normal kidney. After severe AKI, differences of T1-values between the two mouse strains were most pronounced on day 7: this example shows higher T1-values in the ISOM and lower T1-values in the renal cortex of B6 compared to Sv-mice.

For T1 quantification relative values are given comparing the IRI kidney to the contralateral kidney at the different time points. Noteworthy, in contrast to renal perfusion, which was measured only in the cortex (due to a low signal-to-noise ratio on perfusion-maps) the different morphological areas in the kidney can be distinguished easier by T1-mapping and different levels of T1-values in the cortex and the outer and inner stripe of the outer medulla could be shown (Fig 3). When comparing T1-values of the contralateral, non-ischemic kidney (after both, moderate and severe AKI), T1-values of Sv-mice were higher at all time points in the renal cortex and OSOM while T1-values of Sv-mice were lower at all time points in the ISOM (S3 Table).

**Fig 4:**
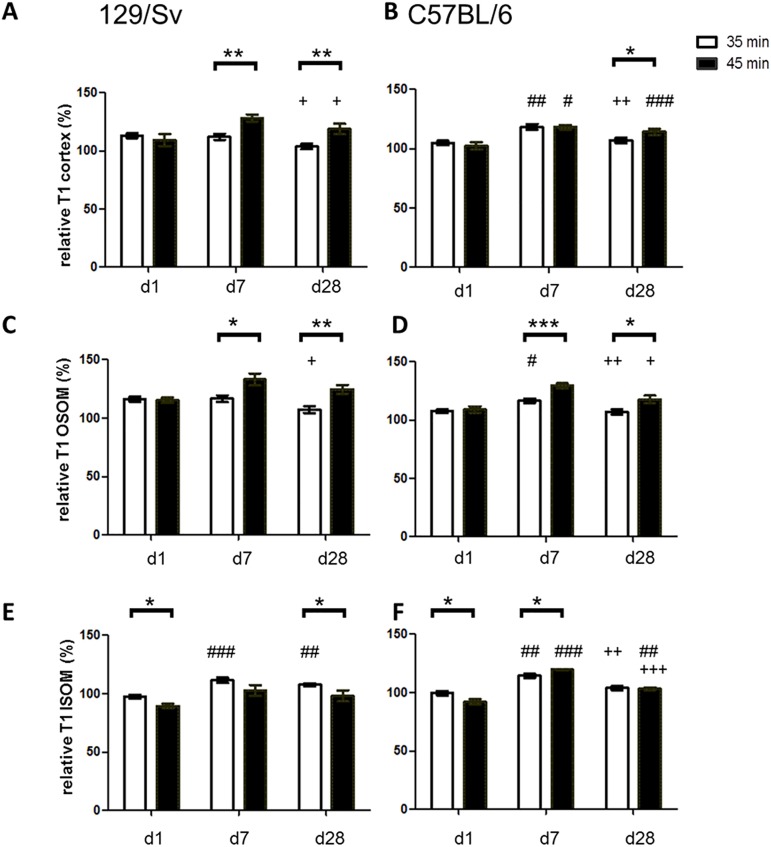
Comparison of T1 values over time. Comparison of relative T1-values after moderate (white bars) vs. severe (black bars) AKI on day 1, 7 and 28 after IRI. Differences were seen in cortex (A, B), OSOM (C, D) as well as ISOM (E, F). Enhanced T1-values were most prominent in severe AKI in both mouse strains in the outer stripe of the outer medulla. * = p<0.05, ** = p<0.01, *** = p<0.001. Differences over time, determined by ANOVA for repeated measurements, are presented as follows: # = p<0.05 vs. day 1, ## = p<0.01 vs. day 1 ### = p<0.001 vs. day 1; + = p<0.05 vs. day7; ++ = p<0.01 vs. day 7, +++ = p<0.001 vs. day 7.

In both mouse strains, no significant difference of T1-values after severe and moderate AKI can be observed in the renal cortex and OSOM on day 1. On day 7 and day 28, T1-values were significantly higher after severe AKI than after moderate AKI in the cortex and OSOM except for cortical T1-values on day 7 in B6-mice (Fig 4). No systematic changes were seen in the ISOM: after moderate AKI, T1-values were higher compared to severe AKI on day 1 in both mouse strains and on day 28 in Sv-mice. On day 7, however, T1-values were higher after severe AKI compared to moderate AKI in B6-mice.

**Fig 5:**
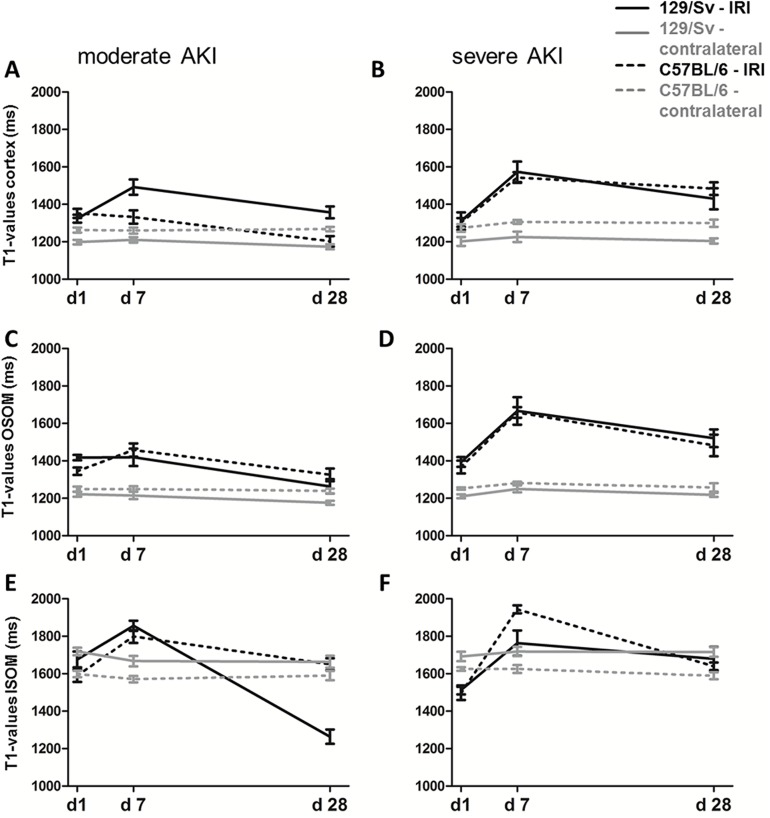
Absolute T1-values of both mouse strains. Comparison of the two mouse strains by T1-mapping. Values of Sv-mice are shown in continuous lines and B6 in dotted lines. Absolute T1-values (ms) of the IRI-kidney are presented in black lines, values of the contralateral kidney are presented in grey. After moderate AKI, Sv-mice had significantly higher T1-values on day 1 in the renal cortex (A) and the OSOM (C). After severe AKI Sv-mice had significantly higher T1-values at day 7 in the cortex (B) and significantly lower values in the ISOM (F) compared to B6 mice pointing towards spatial differences.

When comparing the two mouse strains, differences were found in tissue edema formation mainly in the early phase at d1 or d7 (Fig 5). In moderate AKI (35min) relative T1-relaxation time was higher in the cortex and OSOM of Sv- compared to B6-mice at d1 (p<0.01). In severe AKI (45min IRI) Sv-mice showed higher relative T1-values in the cortex at d7 and lower T1-values in the ISOM at d7 compared to B6-mice. At the endpoint at d28 no significant differences between groups were seen.

### Kidney volume

**Fig 6:**
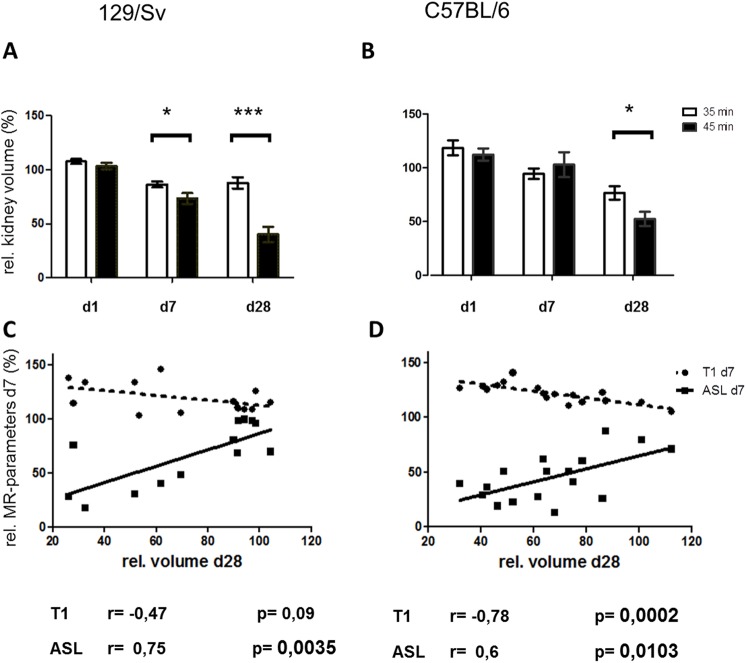
Kidney volume. Comparison of relative kidney volume after moderate (white bars) and severe (black bars) AKI. In both mouse strains severe AKI showed more kidney volume loss (A, B). The correlation of MR-parameters (ASL in continuous line, T1-mapping in dotted line) on day 7 with kidney volume on day 28 is shown in the lower panel (C, D). * = p<0.05, ** = p<0.01, *** = p<0.001.

Already after 7 days there was a difference of relative kidney volume when comparing moderate vs. severe AKI in Sv-mice (86±3% (35min IRI) vs. 73±5% (45min IRI), p<0.05, Fig 6). The difference between moderate and severe AKI increased over time and on day 28 both mouse strains had relative kidney volumes of 40–52% corresponding with severe renal function impairment and CKD (Sv: 88±5% (IRI 35min) vs. 40±7% (IRI 45min), p<0.001; B6: 77±6% (IRI 35min) vs. 52±7% (IRI 45min), p<0.05).

Only in B6 mice, a clear correlation between enhanced T1-values in the OSOM at day 7 and kidney volume loss on day 28 (r = -0.78, p<0.001) could be observed. Relative Perfusion of the renal cortex on day 7 correlated significantly with kidney volume loss in B6- (r = 0.6, p<0.05) as well as in Sv-mice (r = 0.75, p<0.01). Results of correlation analysis are summarized in Table 1.

**Table 1:** Correlation of relative MR-parameters on day 7 after AKI with relative kidney volume loss until day 28.

MR-parameter	region	B6		Sv	
		r =	p =	r =	p =
T1	cortex	ns	ns	0.78	<0.01
	OSOM	0.78	<0.001	ns	ns
	ISOM	0.69	<0.01	0.71	<0.01
ASL	cortex	0.6	<0.05	0.75	<0.01

OSOM = outer stripe of outer medulla, ISOM = inner stripe of outer medulla, ns = not significant.

### Histology

#### Renal fibrosis

**Fig 7:**
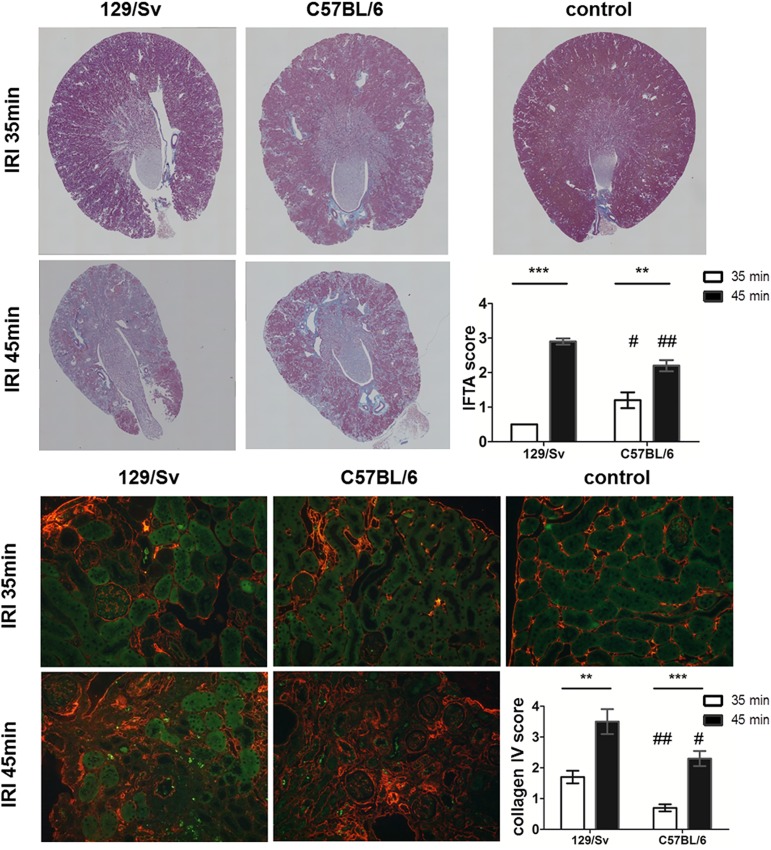
Histology. Masson trichrome stain (upper panels) revealed enhanced interstitial fibrosis after severe AKI due to 45min IRI (whole kidney imaging by Keyence z-stacks). In addition, vast collagen IV deposition was seen after severe AKI but also in cortical focal areas after moderate AKI (lower panels, 200 fold magnification). ** = p<0.01, *** = p<0.001; # = p<0.05 vs. Sv-mice, ## = p<0.01 vs. Sv-mice.

Histology (Masson trichrome, Fig 7 upper rows) showed that renal morphology was almost normal after moderate AKI (35min IRI) at 28 days. After severe AKI increased interstitial fibrosis and tubular atrophy (IFTA) were detected in accordance with the kidney volume loss measured by MRI. In detail, after 35min IRI, only mild changes were found in both mouse strains, according to the IFTA score, while changes were more pronounced in B6- compared to Sv-mice (1.2±0.2 vs. 0.5±0, p<0.05); after 45min IRI, severe (Sv-mice) or moderate (B6-mice) changes were found (2.9±0.1 vs. 2.2±0.2, p<0.01). Collagen IV immunohistochemistry (lower rows) showed that moderate AKI (35min IRI) resulted in focal scaring and severe AKI (45min IRI) showed massive enhancement of connective collagen IV positive tissue. Using the fibrosis score, Sv-mice showed moderate fibrosis and B6-mice showed mild fibrosis after 35min IRI (1.7±0.2 vs. 0.7±0.1, p<0.01); after 45min IRI, very severe (Sv-mice, 3.6±0.5) and moderate (B6-mice, 2.3±0.3, p<0.05) fibrosis were observed.

Relative T1-values on day 7 after IRI correlated significantly with scores from fibrosis in both mouse strains and might therefore have a predictive value. Relative perfusion-values on day 7 after IRI correlated significantly with scores from histology in Sv-mice and with IFTA-scores in B6-mice. Perfusion-values on day 1 after IRI correlated significantly with scores from collagen IV staining in Sv-, but not in B6-mice. No significant correlation of fibrosis markers and T1-values was found on day 1 after IRI. MR-parameters on day 28 after IRI correlated significantly with IFTA-scores in both mouse strains. MR-parameters on day 28 correlated significantly with scores from collagen IV staining in Sv-, but not in B6-mice.

**Fig 8:**
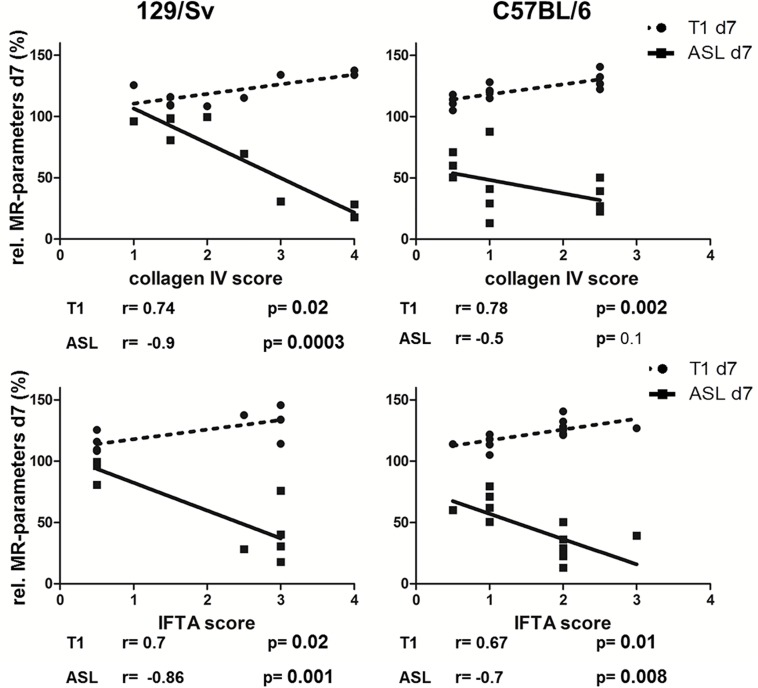
Correlation of MR-parameters on day 7 with histology (collagen IV- and IFTA-score). The correlation of MR-parameters (ASL in continuous line, T1-mapping in dotted line) on day 7 with scores from histology (collagen IV: upper panel, IFTA: lower panel) is shown for Sv- (left column) and B6-mice (right column).

Results of correlation analysis of MR-parameters on day 7 and fibrosis-scores are summarized in Fig 8.

#### Inflammation

**Fig 9:**
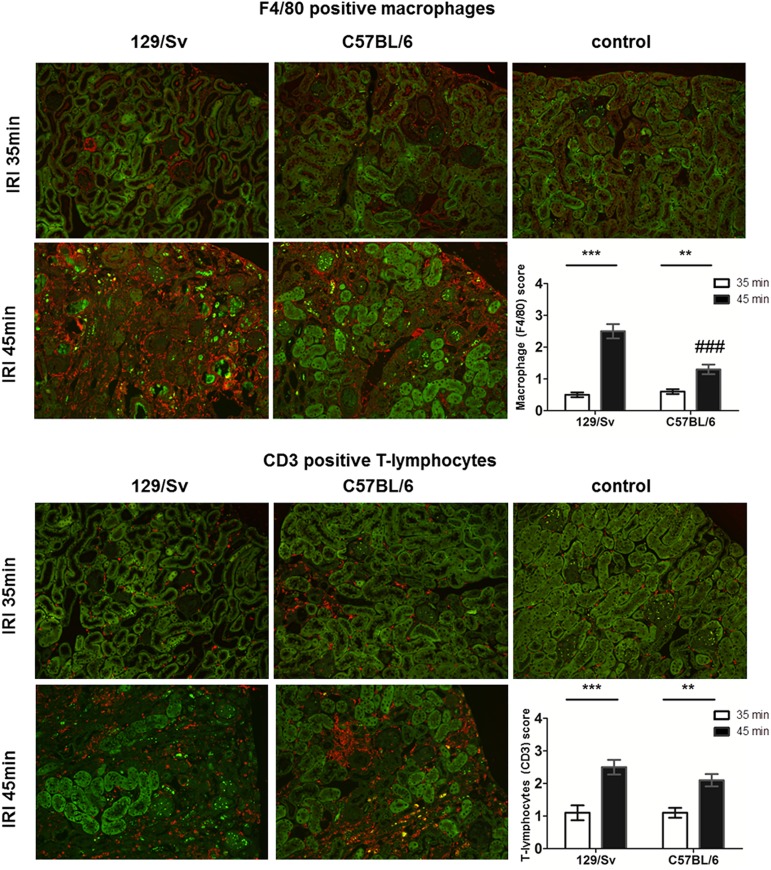
Histology. Monocyte/macrophage staining (F4/80 positive cells, upper panels) revealed minor subcortical infiltrates in areas of small tubular atrophy after 35min IRI. Prolonged IRI caused severe persistent macrophage infiltration in both strains. Sv-mice had more severe infiltrates compared to B6-mice. Inflammatory cells accumulated in areas of tubular atrophy. Similar results were seen with infiltrating T-lymphocytes (CD3 positive cells, lower panels) with enhanced infiltration after 45min IRI. Here no differences between Sv- and B6-mice were detected (200 fold magnification). ** = p<0.01; ### = p<0.001 vs. Sv-mice.

Inflammation with CD3 positive lymphocytes or macrophages were only apparent in small cortical areas with minor tubular atrophy. After 35min IRI, mild inflammatory cell infiltration (macrophages: F4/80 and T-lymphocytes: CD3) was found in both mouse strains (Sv vs. B6; F4/80: 0.5±0.1 vs. 0.6±0.1, ns; CD3: 1.1±0.2 vs 1.1±0.1, ns). Severe inflammatory cell infiltration was seen after prolonged ischemia time. B6-mice showed similar CD3 positive cell infiltrates as Sv-mice (Sv vs. B6: 2.5±1.2 vs. 2.1±0.2, ns) but reduced macrophage infiltration (Sv vs. B6: 2.5±1.2 vs. 1.3±0.2, p<0.001). Examples are depicted in Fig 9.

Relative T1-values on day 7 after IRI correlated significantly with scores from cell infiltration in both mouse strains and might therefore have a predictive value. Relative perfusion-values on day 7 after IRI correlated significantly with scores from histology in both mouse strains. Perfusion-values on day 1 after IRI correlated significantly with scores from CD3 staining in Sv-, but not in B6-mice. No significant correlation of inflammation markers and T1-values was found on day 1 after IRI. MR-parameters on day 28 after IRI correlated significantly with CD3-scores in both mouse strains. MR-parameters on day 28 correlated significantly with scores from F4/80 staining in Sv-, but not in B6-mice.

**Fig 10:**
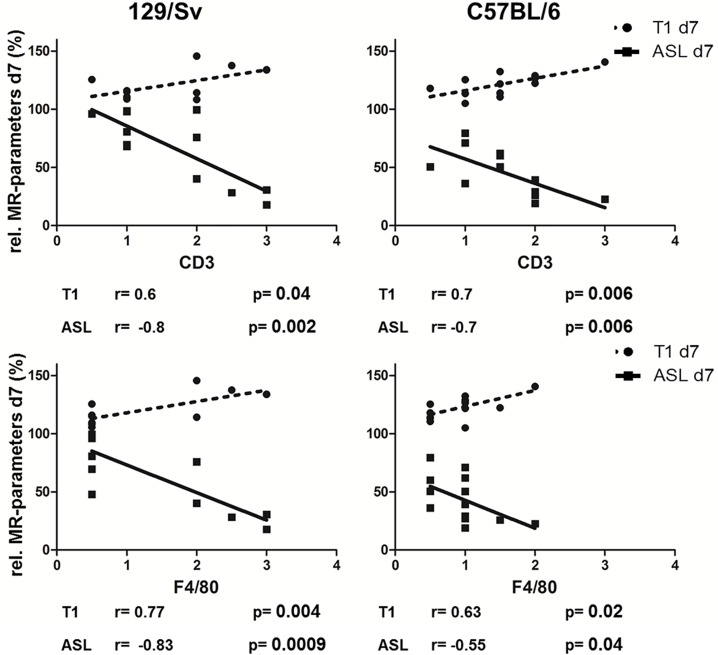
Correlation of MR-parameters on day 7 with histology (CD3 and F4/80 infiltration). The correlation of MR-parameters (ASL in continuous line, T1-mapping in dotted line) on day 7 with scores from histology (CD3: upper panel, F4/80: lower panel) is shown for Sv- (left column) and B6-mice (right column).

Results of correlation analysis of MR-parameters on day 7 and fibrosis-scores are summarized in Fig 10.

## Discussion

Our study showed that functional MRI allows monitoring of renal pathophysiological changes that are caused by IRI. Renal perfusion impairment as well as edema formation both are hallmarks of IRI. These changes could be quantified by MRI, which allowed characterization of strain differences between B6- and Sv-mice after moderate or severe AKI. In both groups, prolonged ischemia time resulting in severe AKI was associated with more severe perfusion impairment and edema formation and consecutively with marked kidney volume loss at d28. A clear correlation between enhanced renal perfusion impairment at d7 and subsequent kidney volume loss until d28 could be shown.

We used ASL for the measurement of renal cortex tissue perfusion, since the accuracy was recently validated in a mouse model, correlating ASL-values with PAH clearance (representing renal plasma flow) [16,21] as well as in a swine model, correlating ASL-values with fluorescent microspheres [22]. Here we compared ASL in the two different mouse strains longitudinally over an observation period of 4 weeks.

We used T1-mapping to investigate renal pathology after AKI, since this parameter was also predictive for AKI severity and further outcome in previous mouse studies [17]. Enhanced T1-values seem to correlate with changes in tissue composition. It has been shown that tissue water content increases T1-values in the kidney and increased tissue water content could be due to cell swelling and capillary leakage after AKI [23]. Additionally, a good correlation of T1-values with histologically proven fibrosis was found previously [24,25].

After moderate AKI, renal perfusion impairment was stronger in B6- compared to Sv-mice at day 7 and day 28. Differences in blood pressure can be responsible for restricted organ perfusion and differences between the two mouse strains concerning blood pressure have been reported earlier: after partial nephrectomy, blood pressure in Sv-mice increased significantly, while blood pressure in B6-animals showed no significant increase [2]. Higher systemic blood pressure could result also in higher renal perfusion. Additionally, a polymorphism for the number of renin genes has been reported; Sv-mice, having two copies of the renin gene (Ren-1^d^ and Ren-2), had tenfold higher plasma renin activity and angiotensin-dependent hypertension, than B6-mice with only one active copy of the renin gene (Ren-1^c^) [26–29]. These characteristics might play a role in the explanation of our result, that Sv-mice have better renal perfusion before and after moderate AKI.

In severe AKI, both mouse strains showed the same trend: in both, lowest values of renal perfusion were found on day 7 with a moderate recovery on day 28, but in both strains, the perfusion level remained impaired compared to the contralateral side (Fig 2). This goes along with results from recent studies on B6-mice, showing that a short ischemia time of 35 min resulted in renal regeneration with only mild scaring and inflammatory cell infiltrates, whereas 45 min IRI resulted in CKD with severe tubulo-interstitial fibrosis, inflammation and impaired renal perfusion [16–18].

Enhanced T1-values at d1 after IRI are more likely to represent tissue edema rather than enhanced connective tissue generation. However, severe renal function impairment might also contribute to less tubular function with anuria. Empty tubules result in less fluid signal possibly also resulting in variation of T1-levels. Since on T1-maps the different areas of the kidney cortex, outer and inner stripe of the outer medulla can be distinguished this method allows to quantify spatial T1-values by using an ROI based analysis. Here we could show that after severe AKI the T1-values were enhanced in the cortex of Sv- compared to B6-mice at day 7. At the same time, the ISOM showed opposite results with Sv-mice showing significantly reduced T1-values. A possible explanation could be that T1-values are reduced because tubules are empty due to impairment of renal function. So far, little attention has been drawn to alterations in the ISOM and the focus has been placed on the OSOM. The T1-maps clearly showed strain characteristic difference in the ISOM as well. It is noteworthy that at four weeks after IRI, B6-mice had significantly less macrophage infiltration than Sv-mice whereas T-lymphocyte expression was similarly enhanced in both strains. The differential role of these two leukocyte subsets in the context of IRI is not clear yet and needs further investigation.

Currently, the tubulo-vascular spatial organization of the vascular bundle that resides in the ISOM is still under debate, due to its complexity. Tubules and vessels in this region are of small caliber [30,31], they are spatially arranged in the vascular bundle and interstitial tissue here is sparse [32,33]. Karlberg and co-workers reported in their study on IRI by clamping the renal artery in rats for 45 min that renal blood flow, measured with labeled microspheres, was most reduced in the ISOM, compared to the other layers [34]. In a study concerning severe AKI in rats by Basile et al., microfil analysis revealed that the reduction in peritubular capillary densitiy was most pronounced in the ISOM compared to OSOM and renal cortex (30–50% reduced compared to sham animals), analyzed 4 weeks after IRI [35]. Assuming that IRI leads to capillary destruction increased T1-values measured in the ISOM might be due to different extent of capillary leakage in the two mouse strains. The exact mechanism in this layer remains incompletely understood.

A recent study by Lu et al. investigating severe AKI after 45min IRI found that B6-mice had more severe F4/80 positive monocyte/macrophage and CD4 positive T-lymphocyte infiltration compared to Sv-mice [4]. A higher grade of cell infiltration, going along with tissue edema, might be another explanation for the higher T1-measures in this layer.

Kidney volume loss due to AKI was similar in both mouse strains and was most prominent after prolonged ischemia time of 45min. A clear correlation between renal perfusion impairment measured by ASL at day 7 and later kidney volume loss after four weeks could be drawn in both mouse strains. Volume loss is associated with irreversible kidney damage and may be interpreted as evidence for progression to CKD. We have shown previously that kidney volume loss in animals of 60% on day 28 after 45 min IRI was associated with severe renal function impairment measured by inulin clearance and severe renal fibrosis[16]. Our findings underline the effect of impaired microcirculation on maladaptive repair and progression to CKD [36].

Correlation of T1-values with kidney volume loss was found in both mouse strains. Knowing that MR-parameters correlate with kidney volume loss four weeks after IRI, prognostic appraisal can be obtained.

Until now, characterization of mouse strains has mainly been performed as end-point measurements at varying time points. With functional MRI, parameters can be obtained in vivo, noninvasively and pathophysiological changes can be studied over time. Longitudinal assessment of renal changes would be most useful in pre-clinical drug testing studies since the amount of animals needed in individual studies could be reduced. Oftentimes the mode of action of specific therapies is not clear and animals are sacrificed at different time points to examine by histology whether the tested compound has an effect. Functional MRI studies could help identify the optimal time point for further analysis.

The MRI-examination takes approximately 30 minutes and different parameters can be assessed in one MRI session in the individual mouse.

Our results showed some differences between the two mouse strains in the susceptibility to IRI. Characterization of these differences is useful for understanding, planning and interpretation of experimental studies. The findings stress the need of proper controls in experimental animal studies.

Limitation of our study is that we did not determine baseline values for T1-mapping and ASL. Relative values were calculated in relation to the contralateral, non-ischemic kidney. We did not consider changes of the contralateral side however it has been shown that the contralateral kidney increases in kidney volume after moderate and severe AKI by approximately 20% by day 28 after AKI [16]. Therefore, the relative differences calculated in this study might overestimate the changes in kidney volume after IRI of the single kidney. Concerning renal perfusion and T1-mapping no major changes of the contralateral kidney have been reported previously over time after IRI [16,17].

In conclusion, we showed that by MRI strain differences between B6- and Sv-mice at different time points are present.

T1-mapping and ASL can distinguish between different severities of AKI showing more pronounced renal perfusion impairment and interstitial edema formation after prolonged ischemia time of 45min. In the early period after AKI, ASL-values correlate with kidney volume on day 28 in both mouse strains and therefore could serve as a predictive marker for renal outcome.

Functional MRI is useful to characterize severity and susceptibility to ischemia induced AKI, thus facilitating planning of animal studies.

## Supporting information

S1 Table Perfusion-values of the renal cortex, measured by ASL on day 1, day 7 and day 28 after 35 min and 45 min ischemia reperfusion injury. Values are given in ml/min/100g for the IRI kidney and the non-ischemic contralateral control kidney (control). Values of relative Perfusion (Rel. value) are given in percent. SEM = standard error of the mean. min = minutes.
(DOCX)

S2 Table T1-values of the renal cortex, the outer stripe of the outer medulla and the inner stripe of the outer medulla, measured by T1-mapping on day 1, day 7 and day 28 after 35 min and 45 min ischemia reperfusion injury. Values are given in ms for the IRI kidney (IRI) and the non-ischemic contralateral control kidney (control). Values of relative T1-times (Rel. value) are given in percent. SEM = standard error of the mean, min = minutes.
(DOCX)

S3 Table Comparison of T1-values of the contralateral, non-ischemic kidney (after both, moderate and severe AKI). Mean values (±standard error of the mean) are given in ms.
(DOCX)
