# Supplementary material for: Functional MRI for characterization of renal perfusion impairment and edema formation due to acute kidney injury in different mouse strains
Source: PLoS One. 2017 Mar 20;12(3):e0173248. doi: 10.1371/journal.pone.0173248 (PMC5358739; doi:10.1371/journal.pone.0173248)
Supplement: S1 Table — Values are given in ml/min/100g for the IRI kidney and the non-ischemic contralateral control kidney (control). Values of relative Perfusion (Rel. value) are given in percent. SEM = standard error of the mean. min = minutes. (DOCX) [file pone.0173248.s001.docx]

**S 1 Table.** Perfusion-values of the renal cortex, measured by ASL on day 1, day 7 and day 28 after 35 min and 45 min ischemia reperfusion injury.

| IRI 35 min | | | | | | | | | | |
| --- | --- | --- | --- | --- | --- | --- | --- | --- | --- | --- |
|  | Day 1 | | | Day 7 | | | | Day 28 | | |
|  | IRI | control | Rel. value | IRI | | control | Rel. value | IRI | control | Rel. value |
| 129/Sv | 615 | 969 | 63 | 429 | | 979 | 44 | 812 | 831 | 98 |
|  | 661 | 924 | 72 | 854 | | 847 | 101 | 1123 | 906 | 124 |
|  | 459 | 1006 | 46 | 756 | | 991 | 76 | 803 | 790 | 102 |
|  | 581 | 972 | 60 | 1049 | | 1014 | 103 | 1121 | 1089 | 103 |
|  | 544 | 813 | 67 | 597 | | 839 | 71 | 835 | 854 | 98 |
|  | 438 | 790 | 55 | 892 | | 860 | 104 | 785 | 749 | 105 |
|  | 618 | 851 | 73 | 705 | | 713 | 99 | 1015 | 1007 | 101 |
|  | 728 | 866 | 84 | 643 | | 1055 | 61 | 1258 | 1231 | 102 |
|  | 992 | 873 | 114 | - | | - |  | 950 | 1095 | 87 |
| mean value | **626** | **896** | **70** | **741** | | **912** | **82** | **967** | **950** | **102** |
| SEM | 52 | 24 | 6 | 64 | | 38 | 8 | 54 | 51 | 3 |
|  | | | | | | | | | | |
| C57BL/6 | 260 | 533 | 49 | 269 | 434 | | 62 | 309 | 551 | 56 |
|  | 299 | 544 | 55 | 275 | 543 | | 51 | 407 | 373 | 109 |
|  | 370 | 565 | 66 | 199 | 331 | | 60 | 401 | 414 | 97 |
|  | 318 | 600 | 53 | 314 | 395 | | 80 | 431 | 548 | 79 |
|  | 370 | 512 | 72 | 488 | 686 | | 71 | 769 | 804 | 96 |
|  | 229 | 573 | 40 | 203 | 400 | | 51 | 436 | 589 | 74 |
|  | 368 | 454 | 81 | 60 | 459 | | 13 | 326 | 431 | 76 |
|  | 410 | 618 | 66 | 291 | 331 | | 88 | 390 | 632 | 62 |
|  | 340 | 511 | 67 | 149 | 363 | | 41 | 443 | 552 | 80 |
|  | 284 | 376 | 75 | 223 | 614 | | 36 | 205 | 592 | 35 |
| mean value | **325** | **529** | **62** | **247** | **456** | | **55** | **412** | **549** | **76** |
| SEM | 18 | 23 | 4 | 36 | 38 | | 7 | 46 | 39 | 7 |
|  | | | | | | | | | | |
| IRI 45 min | | | | | | | | | | |
|  | Day 1 | | | Day 7 | | | | Day 28 | | |
| 129/Sv | IK | NIK | Rel. value | IK | NIK | | Rel. value | IK | NIK | Rel. value |
|  | 695 | 752 | 92 | 218 | 812 | | 27 | 548 | 837 | 65 |
|  | 820 | 881 | 93 | 112 | 672 | | 17 | 560 | 919 | 61 |
|  | 892 | 1023 | 87 | 256 | 935 | | 27 | 896 | 1047 | 86 |
|  | 350 | 1040 | 34 | 736 | 1321 | | 56 | 782 | 956 | 82 |
|  | 646 | 1007 | 64 | 418 | 1096 | | 38 | 864 | 1002 | 86 |
| mean value | **680** | **941** | **74** | **348** | **967** | | **33** | **730** | **952** | **76** |
| SEM | 93 | 55 | 11 | 109 | 113 | | 8 | 74 | 36 | 5 |
|  | | | | | | | | | | |
| C57BL/6 | 414 | 628 | 66 | 150 | 663 | | 23 | 69 | 504 | 14 |
|  | 436 | 613 | 71 | 118 | 456 | | 26 | 393 | 478 | 82 |
|  | 411 | 650 | 63 | 183 | 464 | | 39 | 98 | 373 | 26 |
|  | 447 | 694 | 64 | 113 | 596 | | 19 | 257 | 487 | 53 |
|  | 397 | 564 | 70 | 259 | 513 | | 51 | 300 | 433 | 69 |
|  | 408 | 612 | 67 | 138 | 508 | | 27 | 372 | 447 | 83 |
|  | 448 | 691 | 65 | 144 | 491 | | 29 | 186 | 392 | 47 |
| mean value | **423** | **636** | **67** | **158** | **527** | | **31** | **239** | **445** | **54** |
| SEM | 8 | 17 | 1 | 19 | 29 | | 4 | 48 | 19 | 10 |

Values are given in ml/min/100g for the IRI kidney and the non-ischemic contralateral control kidney (control). Values of relative Perfusion (Rel. value) are given in percent. SEM=standard error of the mean. min=minutes.
