# Supplementary material for: Functional MRI for characterization of renal perfusion impairment and edema formation due to acute kidney injury in different mouse strains
Source: PLoS One. 2017 Mar 20;12(3):e0173248. doi: 10.1371/journal.pone.0173248 (PMC5358739; doi:10.1371/journal.pone.0173248)
Supplement: S2 Table — Values are given in ms for the IRI kidney (IRI) and the non-ischemic contralateral control kidney (control). Values of relative T1-times (Rel. value) are given in percent. SEM = standard error of the mean, min = minutes. (DOCX) [file pone.0173248.s002.docx]

**S 2 Table.** T1-values of the renal cortex, the outer stripe of the outer medulla and the inner stripe of the outer medulla, measured by T1-mapping on day 1, day 7 and day 28 after 35 min and 45 min ischemia reperfusion injury.

Panel A: Day 1:

| IRI 35 min | | | | | | | | | |
| --- | --- | --- | --- | --- | --- | --- | --- | --- | --- |
|  | IRI kidney | | | control kidney | | | Rel. value | | |
|  | Cortex | OSOM | ISOM | Cortex | OSOM | ISOM | Cortex | OSOM | ISOM |
| 129/Sv | 1315 | 1411 | 1862 | 1126 | 1168 | 1761 | 117 | 121 | 106 |
|  | 1412 | 1452 | 1714 | 1178 | 1218 | 1745 | 120 | 119 | 98 |
|  | 1411 | 1414 | 1787 | 1176 | 1209 | 1756 | 120 | 117 | 102 |
|  | 1380 | 1406 | 1732 | 1238 | 1267 | 1749 | 111 | 111 | 99 |
|  | 1441 | 1480 | 1690 | 1211 | 1196 | 1663 | 119 | 124 | 102 |
|  | 1332 | 1352 | 1732 | 1253 | 1301 | 1822 | 106 | 104 | 95 |
|  | 1262 | 1426 | 1515 | 1203 | 1225 | 1647 | 105 | 116 | 92 |
|  | 1265 | 1363 | 1480 | 1211 | 1224 | 1646 | 104 | 111 | 90 |
|  | 1366 | 1460 | 1576 | 1189 | 1192 | 1685 | 115 | 122 | 94 |
| mean value | **1354** | **1418** | **1676** | **1198** | **1222** | **1719** | **113** | **116** | **97** |
| SEM | 26 | 17 | 52 | 15 | 16 | 25 | 3 | 3 | 2 |
|  | | | | | | | | | |
| C57BL/6 | 1313 | 1332 | 1562 | 1227 | 1171 | 1612 | 107 | 114 | 97 |
|  | 1317 | 1377 | 1722 | 1312 | 1226 | 1594 | 100 | 112 | 108 |
|  | 1350 | 1389 | 1672 | 1256 | 1315 | 1615 | 107 | 106 | 104 |
|  | 1370 | 1378 | 1709 | 1257 | 1297 | 1660 | 109 | 106 | 103 |
|  | 1248 | 1234 | 1479 | 1181 | 1210 | 1477 | 106 | 102 | 100 |
|  | 1392 | 1421 | 1628 | 1314 | 1301 | 1614 | 106 | 109 | 101 |
|  | 1403 | 1378 | 1565 | 1271 | 1230 | 1547 | 110 | 112 | 101 |
|  | 1353 | 1371 | 1614 | 1224 | 1252 | 1605 | 111 | 110 | 101 |
|  | 1300 | 1325 | 1561 | 1296 | 1229 | 1664 | 100 | 108 | 94 |
|  | 1191 | 1239 | 1382 | 1292 | 1256 | 1595 | 92 | 99 | 87 |
| mean value | **1324** | **1344** | **1589** | **1263** | **1249** | **1598** | **105** | **108** | **99** |
| SEM | 27 | 26 | 43 | 18 | 18 | 22 | 2 | 2 | 2 |
|  | | | | | | | | | |
| IRI 45 min | | | | | | | | | |
|  | IRI kidney | | | control kidney | | | Rel. Value | | |
|  | Cortex | OSOM | ISOM | Cortex | OSOM | ISOM | Cortex | OSOM | ISOM |
| 129/Sv | 1171 | 1305 | 1419 | 1167 | 1184 | 1683 | 100 | 110 | 84 |
|  | 1307 | 1404 | 1541 | 1218 | 1216 | 1701 | 107 | 115 | 91 |
|  | 1259 | 1373 | 1526 | 1288 | 1243 | 1666 | 98 | 110 | 92 |
|  | 1431 | 1458 | 1540 | 1153 | 1191 | 1629 | 124 | 122 | 95 |
|  | 1387 | 1429 | 1540 | 1184 | 1219 | 1783 | 117 | 117 | 86 |
| mean value | **1311** | **1394** | **1513** | **1202** | **1211** | **1692** | **109** | **115** | **89** |
| SEM | 42 | 24 | 22 | 22 | 10 | 23 | 5 | 2 | 2 |
|  | | | | | | | | | |
| C57BL/6 | 1276 | 1298 | 1448 | 1250 | 1254 | 1625 | 102 | 104 | 89 |
|  | 1235 | 1253 | 1337 | 1346 | 1254 | 1630 | 92 | 100 | 82 |
|  | 1356 | 1397 | 1477 | 1261 | 1247 | 1630 | 108 | 112 | 91 |
|  | 1333 | 1479 | 1595 | 1177 | 1241 | 1622 | 113 | 119 | 98 |
|  | 1217 | 1274 | 1467 | 1283 | 1231 | 1665 | 95 | 103 | 88 |
|  | 1321 | 1428 | 1583 | 1296 | 1255 | 1582 | 102 | 114 | 100 |
|  | 1384 | 1440 | 1561 | 1306 | 1281 | 1622 | 106 | 112 | 96 |
| mean value | **1303** | **1367** | **1495** | **1274** | **1252** | **1625** | **102** | **109** | **92** |
| SEM | 25 | 37 | 37 | 22 | 6 | 10 | 3 | 3 | 3 |

Panel B: Day 7:

| IRI 35 min | | | | | | | | | |
| --- | --- | --- | --- | --- | --- | --- | --- | --- | --- |
|  | IRI kidney | | | control kidney | | | Rel. Value | | |
|  | Cortex | OSOM | ISOM | Cortex | OSOM | ISOM | Cortex | OSOM | ISOM |
| 129/Sv | 1230 | 1278 | 1791 | 1161 | 1182 | 1643 | 106 | 108 | 109 |
|  | 1390 | 1395 | 1907 | 1172 | 1170 | 1644 | 119 | 119 | 116 |
|  | 1283 | 1364 | 1928 | 1179 | 1127 | 1534 | 109 | 121 | 126 |
|  | 1261 | 1298 | 1843 | 1168 | 1164 | 1599 | 108 | 112 | 115 |
|  | 1280 | 1325 | 1899 | 1220 | 1249 | 1751 | 105 | 106 | 108 |
|  | 1267 | 1345 | 1954 | 1241 | 1243 | 1781 | 102 | 108 | 110 |
|  | 1507 | 1501 | 1815 | 1244 | 1250 | 1640 | 121 | 120 | 111 |
|  | 1440 | 1650 | 1696 | 1230 | 1249 | 1643 | 117 | 132 | 103 |
|  | 1573 | 1617 | 1875 | 1282 | 1308 | 1774 | 123 | 124 | 106 |
| mean value | **1359** | **1419** | **1856** | **1211** | **1216** | **1668** | **112** | **117** | **112** |
| SEM | 50 | 56 | 33 | 17 | 23 | 34 | 3 | 4 | 3 |
|  | | | | | | | | | |
| C57BL/6 | 1514 | 1431 | 1760 | 1191 | 1173 | 1483 | 127 | 122 | 119 |
|  | 1370 | 1467 | 1816 | 1232 | 1243 | 1620 | 111 | 118 | 112 |
|  | 1383 | 1414 | 1735 | 1247 | 1240 | 1592 | 111 | 114 | 109 |
|  | 1468 | 1394 | 1862 | 1240 | 1227 | 1607 | 118 | 114 | 116 |
|  | 1281 | 1252 | 1616 | 1212 | 1191 | 1575 | 106 | 105 | 103 |
|  | 1483 | 1394 | 1695 | 1258 | 1259 | 1480 | 118 | 111 | 115 |
|  | 1692 | 1582 | 1798 | 1364 | 1303 | 1574 | 124 | 121 | 114 |
|  | 1546 | 1439 | 1820 | 1252 | 1250 | 1539 | 123 | 115 | 118 |
|  | 1679 | 1567 | 1878 | 1302 | 1303 | 1614 | 129 | 120 | 116 |
|  | 1504 | 1642 | 2006 | 1303 | 1308 | 1626 | 115 | 126 | 123 |
| mean value | **1492** | **1458** | **1799** | **1260** | **1250** | **1571** | **118** | **117** | **114** |
| SEM | 53 | 46 | 44 | 21 | 19 | 22 | 3 | 2 | 2 |
|  | | | | | | | | | |
| IRI 45 min | | | | | | | | | |
|  | IRI kidney | | | control kidney | | | Rel. Value | | |
|  | Cortex | OSOM | ISOM | Cortex | OSOM | ISOM | Cortex | OSOM | ISOM |
| 129/Sv | 1602 | 1764 | 1808 | 1303 | 1316 | 1752 | 123 | 134 | 103 |
|  | 1520 | 1664 | 1721 | 1219 | 1242 | 1647 | 125 | 134 | 104 |
|  | 1723 | 1735 | 1789 | 1247 | 1260 | 1688 | 138 | 138 | 106 |
|  | 1397 | 1383 | 1543 | 1132 | 1209 | 1789 | 123 | 114 | 86 |
|  | 1626 | 1788 | 1959 | 1232 | 1226 | 1718 | 132 | 146 | 114 |
| mean value | **1574** | **1667** | **1764** | **1227** | **1251** | **1719** | **128** | **133** | **103** |
| SEM | 50 | 67 | 62 | 25 | 17 | 22 | 3 | 5 | 4 |
|  | | | | | | | | | |
| C57BL/6 | 1553 | 1808 | 2052 | 1341 | 1285 | 1725 | 116 | 141 | 119 |
|  | 1629 | 1592 | 1906 | 1298 | 1300 | 1611 | 126 | 122 | 118 |
|  | 1504 | 1656 | 1934 | 1308 | 1304 | 1635 | 115 | 127 | 118 |
|  | 1532 | 1611 | 1884 | 1268 | 1249 | 1576 | 121 | 129 | 120 |
|  | 1498 | 1696 | 1968 | 1314 | 1280 | 1651 | 114 | 133 | 119 |
|  | 1652 | 1640 | 1953 | 1339 | 1294 | 1621 | 123 | 127 | 120 |
|  | 1434 | 1609 | 1912 | 1278 | 1255 | 1559 | 112 | 128 | 123 |
| mean value | **1543** | **1659** | **1944** | **1307** | **1281** | **1625** | **118** | **130** | **120** |
| SEM | 31 | 30 | 23 | 11 | 9 | 22 | 2 | 2 | 1 |

Panel C: Day 28:

| IRI 35 min | | | | | | | | | |
| --- | --- | --- | --- | --- | --- | --- | --- | --- | --- |
|  | IRI kidney | | | control kidney | | | Rel. Value | | |
|  | Cortex | OSOM | ISOM | Cortex | OSOM | ISOM | Cortex | OSOM | ISOM |
| 129/Sv | 1160 | 1176 | 1741 | 1134 | 1171 | 1610 | 102 | 100 | 108 |
|  | 1180 | 1192 | 1692 | 1186 | 1158 | 1698 | 99 | 103 | 100 |
|  | 1143 | 1175 | 1820 | 1154 | 1137 | 1713 | 99 | 103 | 106 |
|  | 1184 | 1217 | 1768 | 1226 | 1228 | 1630 | 97 | 99 | 108 |
|  | 1114 | 1144 | 1673 | 1137 | 1146 | 1482 | 98 | 100 | 113 |
|  | 1193 | 1291 | 1868 | 1171 | 1201 | 1804 | 102 | 107 | 104 |
|  | 1300 | 1315 | 1850 | 1140 | 1145 | 1735 | 114 | 115 | 107 |
|  | 1347 | 1491 | 1909 | 1169 | 1196 | 1719 | 115 | 125 | 111 |
|  | 1333 | 1375 | 1760 | 1245 | 1206 | 1585 | 107 | 114 | 111 |
| mean value | **1217** | **1264** | **1787** | **1174** | **1176** | **1664** | **104** | **107** | **108** |
| SEM | 35 | 47 | 33 | 16 | 13 | 39 | 3 | 4 | 2 |
|  | | | | | | | | | |
| C57BL/6 | 1273 | 1259 | 1395 | 1226 | 1189 | 1527 | 104 | 106 | 91 |
|  | 1307 | 1314 | 1773 | 1283 | 1269 | 1668 | 102 | 104 | 106 |
|  | 1341 | 1323 | 1726 | 1314 | 1270 | 1674 | 102 | 104 | 103 |
|  | 1279 | 1211 | 1679 | 1235 | 1229 | 1654 | 104 | 99 | 102 |
|  | 1214 | 1195 | 1643 | 1193 | 1158 | 1583 | 102 | 103 | 104 |
|  | 1407 | 1306 | 1643 | 1293 | 1247 | 1579 | 109 | 105 | 104 |
|  | 1488 | 1423 | 1665 | 1296 | 1289 | 1661 | 115 | 110 | 100 |
|  | 1331 | 1308 | 1632 | 1270 | 1232 | 1428 | 105 | 106 | 114 |
|  | 1409 | 1355 | 1639 | 1293 | 1255 | 1556 | 109 | 108 | 105 |
|  | 1524 | 1566 | 1713 | 1277 | 1255 | 1567 | 119 | 125 | 109 |
| mean value | **1357** | **1326** | **1651** | **1268** | **1239** | **1590** | **107** | **107** | **104** |
| SEM | 40 | 44 | 41 | 15 | 16 | 32 | 2 | 3 | 2 |
|  | | | | | | | | | |
| IRI 45 min | | | | | | | | | |
|  | IRI kidney | | | control kidney | | | Rel. Value | | |
|  | Cortex | OSOM | ISOM | Cortex | OSOM | ISOM | Cortex | OSOM | ISOM |
| 129/Sv | 1486 | 1586 | 1866 | 1229 | 1231 | 1689 | 121 | 129 | 110 |
|  | 1559 | 1564 | 1715 | 1223 | 1243 | 1750 | 127 | 126 | 98 |
|  | 1377 | 1421 | 1577 | 1230 | 1232 | 1760 | 112 | 115 | 90 |
|  | 1492 | 1636 | 1500 | 1159 | 1207 | 1757 | 129 | 136 | 85 |
|  | 1238 | 1398 | 1739 | 1182 | 1182 | 1626 | 105 | 118 | 107 |
| mean value | **1430** | **1521** | **1679** | **1205** | **1219** | **1716** | **119** | **125** | **98** |
| SEM | 51 | 43 | 59 | 13 | 10 | 24 | 4 | 3 | 4 |
|  | | | | | | | | | |
| C57BL/6 | 1561 | 1691 | 1737 | 1377 | 1357 | 1596 | 113 | 125 | 109 |
|  | 1347 | 1244 | 1562 | 1260 | 1186 | 1511 | 107 | 105 | 103 |
|  | 1602 | 1630 | 1646 | 1333 | 1298 | 1656 | 120 | 126 | 99 |
|  | 1491 | 1456 | 1610 | 1220 | 1187 | 1551 | 122 | 123 | 104 |
|  | 1400 | 1446 | 1637 | 1328 | 1269 | 1638 | 105 | 114 | 100 |
|  | 1458 | 1370 | 1638 | 1296 | 1262 | 1585 | 113 | 109 | 103 |
|  | 1529 | 1542 | 1648 | 1283 | 1248 | 1586 | 119 | 124 | 104 |
| mean value | **1484** | **1483** | **1640** | **1300** | **1258** | **1589** | **114** | **118** | **103** |
| SEM | 37 | 62 | 21 | 21 | 25 | 20 | 3 | 3 | 1 |

Values are given in ms for the IRI kidney (IRI) and the non-ischemic contralateral control kidney (control). Values of relative T1-times (Rel. value) are given in percent. SEM=standard error of the mean, min=minutes.
