# Supplementary material for: Functional MRI for characterization of renal perfusion impairment and edema formation due to acute kidney injury in different mouse strains
Source: PLoS One. 2017 Mar 20;12(3):e0173248. doi: 10.1371/journal.pone.0173248 (PMC5358739; doi:10.1371/journal.pone.0173248)
Supplement: S3 Table — Mean values (±standard error of the mean) are given in ms. (DOCX) [file pone.0173248.s003.docx]

**S 3 Table. Comparison of T1 values of the contralateral, non-ischemic kidney (after both, moderate and severe AKI).**

|  | Day 1 | | | Day 7 | | | Day 28 | | |
| --- | --- | --- | --- | --- | --- | --- | --- | --- | --- |
|  | Sv | B6 | p-value | Sv | B6 | p-value | Sv | B6 | p-value |
| cortex | 1200±11 | 1268±11 | <0.001 | 1216±13 | 1279±12 | <0.01 | 1129±32 | 1156±69 | ns |
| OSOM | 1218±9 | 1250±9 | <0.05 | 1508±50 | 1541±34 | ns | 1090±37 | 1064±68 | ns |
| ISOM | 1710±16 | 1609±11 | <0.001 | 1686±21 | 1593±14 | <0.001 | 1683±23 | 1589±16 | <0.05 |

Mean values (±standard error of the mean) are given in ms.
